# Guiding a Diclofenac Sodium Dual-Release Sustained Formulation Development Through In Vitro–In Vivo Relationship Based on Physiologically Based Pharmacokinetics

**DOI:** 10.3390/pharmaceutics18050613

**Published:** 2026-05-18

**Authors:** Qizheng Wang, Pengcheng Guo, Tianci Hu, Longjie Li, Tingxi Zhu, Yue Pan, Xiaoqiang Xiang, Jianxin Wang

**Affiliations:** 1 School of Pharmaceutical Sciences, Fudan University, Key Laboratory of Smart Drug Delivery, Ministry of Education & National Key Laboratory of Advanced Drug Formulations for Overcoming Delivery Barriers, Shanghai 201203, China; pharma_wang@126.com (Q.W.); 20111030046@fudan.edu.cn (P.G.); hutiancicpu@163.com (T.H.); lilj23@m.fudan.edu.cn (L.L.); 22301170103@m.fudan.edu.cn (T.Z.); panyue1107@126.com (Y.P.); xiangxq@fudan.edu.cn (X.X.); 2 Department of Advanced Formulations, Quzhou Fudan Institute, Quzhou 324002, China; 3 The Second People’s Hospital of Quzhou, Quzhou 324022, China

**Keywords:** PBPK, IVIVR, Diclofenac sodium, dual-release sustained formulation, Gastroplus^TM^, bioequivalence

## Abstract

**Background:** Dual-release sustained formulations enable rapid drug release for prompt therapeutic onset while retaining the characteristics of sustained-release dosage forms. However, due to the complexity of this dosage form, conventional trial-and-error approaches fail to mitigate development risks or improve the success rate of bioequivalence studies of the generic product. Accordingly, the present study aims to investigate the feasibility of guiding a dual-release generic formulation screening through establishing a quality-related media condition through construction of PBPK models and IVIVR for the reference product. **Methods:** Here, Difene^®^ was selected as the reference product, and GastroPlus^TM^ was employed as the simulation platform. Pharmacokinetic data obtained from the literature and in vitro dissolution test results were integrated to construct the PBPK model for the reference product and establish IVIVR in different media, respectively. A quality-related media condition was determined for formulation screening of the generic product. A pharmacokinetic study in beagle dogs was then conducted to evaluate the bioequivalence between the generic and the reference product. **Results:** In the PBPK modeling and IVIVR study, the PBPK model was successfully established. The IVIVR for the pH 4.0–pH 6.0–pH 6.8 media was optimal in all media conditions, with fold error ratios of 1.11, 0.86, and 1.11 for C_max_, AUC, and T_max_, respectively, all falling within the 0.80–1.25 range. Employing this medium as the quality-related media, the optimized generic product exhibited an f2 factor of 76 with the reference product in vitro. Pharmacokinetic studies in beagle dogs demonstrated that the geometric mean ratios and 90% confidence intervals for AUC and C_max_ of the generic product versus the reference product were within the 80.0–125.0% range. No statistically significant difference was observed for T_max_, indicating bioequivalence between the two products. **Conclusions:** Overall, our study provides a strategic approach for generic development and a novel research framework for the generic development of other dual-release formulations.

## 1. Introduction

As a specialized form of modified-release formulation, dual-release sustained formulations can be designed for the chronological characteristics of disease and integrating the physicochemical properties of the drug substance with its absorption profile in vivo, thereby enabling synergistic interaction between immediate-release and sustained-release components to enhance therapeutic efficacy and fulfill specific clinical requirements [1,2].

However, due to the formulation characteristics, it precisely increases the complexity of generic development, particularly for the multiple-unit pellet system (MUPS). Conventional generic development relies primarily on trial-and-error approaches, wherein material ratios and weight gains of coating are adjusted and optimized to achieve curve fitting with the reference product in various dissolution media conditions, thereby increasing the probability of success in bioequivalence studies. This approach is associated with high trial-and-error costs and significant risks. Therefore, establishing quality-related dissolution conditions through PBPK (physiologically based pharmacokinetic) modeling and IVIVR (in vitro and in vivo relationship) analysis has become increasingly critical for guiding formulation development and mitigating risks in the generic development of dual-release sustained-release products [3,4,5].

PBPK models are constructed through the integration of drug physicochemical properties, formulation characteristics, and physiological, biochemical, and anatomical parameters of the organism [6,7,8]. By treating each tissue or organ as a compartment connected via blood flow [9,10], PBPK models simulate the kinetic processes of drug absorption, distribution, metabolism, and excretion (A.D.M.E) in biological tissues, as well as concentration–time profiles, thereby predicting drug absorption and metabolic processes in vivo [5]. Currently, the application of PBPK models, predicting pharmacokinetic behavior in animals, humans and diverse populations (age, ethnicity, etc.) through drug–drug interactions, has been widely adopted in new drug development [11,12,13] and regulatory review [14,15,16,17].

Among the various commercial pharmacokinetic software platforms available, GastroPlus^TM^ is a commonly used simulation and modeling platform [18]. It can establish a PBPK model by integrating pharmacokinetic data from intravenous administration, immediate-release formulations, or enteric-coated formulations, utilizing the advanced compartmental absorption and transit (ACAT) model as the absorption model [19]. Prediction capability for oral drug absorption can be enhanced through incorporation of multiple absorption and metabolic factors [20,21]. Furthermore, the software enables establishing linear or nonlinear correlations between in vitro drug release and in vivo absorption through an IVIVR based on a PBPK model [22,23,24]. By calculating the fold error ratio of key pharmacokinetic parameters, in vivo pharmacokinetic processes can be predicted from in vitro dissolution data.

Difene^®^ (Diclofenac sodium dual-release enteric-coated capsule), a classic dual-release formulation product, comprises enteric-coated pellets and sustained-release pellets. The enteric-coated pellets minimize gastric irritation from the active ingredient while ensuring rapid drug release in the intestine, enabling rapid entry of Diclofenac sodium into the systemic circulation, whereas the sustained-release pellets maintain prolonged release of Diclofenac sodium. The combination of these two pellet types generates favorable pharmacokinetic characteristics [25]. The product was approved in China in 2001. Currently, no generic product is approved in the domestic market of China, and no studies involving the application of PBPK models or IVIVR to guide formulation development were published in the field of generic development.

In this study, Difene^®^ was chosen as a reference drug, and GastroPlus^TM^ (version 9.8.3) (Simulation Plus, Inc., Research Triangle Park, NC, USA) was used as a simulation tool to establish a PBPK model and IVIVR of the reference product. Quality-related media condition was validated through IVIVR and was used for formulation screening of the generic product. Pharmacokinetic studies in beagle dogs were performed to validate the bioequivalence of the reference and the generic product, and the feasibility of using a PBPK model to guide the generic development of dual-release sustained formulation.

## 2. Materials and Methods

### 2.1. Reagents, Chemicals and Animals

The reference standard, Diclofenac sodium (100%), was purchased from the National Institutes for Food and Drug (Beijing, China). The internal standard (IS), Indometacin (98%), was purchased from Meilunbio Co., Ltd. (Dalian, China). Difene^®^ 75 mg capsules (Temmler Ireland Ltd., Killorglin, Ireland) were purchased commercially from the Chinese market. Methanol was purchased from Fisher Chemical (Seoul, Republic of Korea), acetonitrile was purchased from Sigma-Aldrich (Schnelldorf, Germany) and formic acid was purchased from TCI Shanghai Co., Ltd. (Shanghai, China) and they were all of HPLC grade. Hydrochloric acid, sodium hydroxide, acetic acid, phosphoric acid, potassium dihydrogen phosphate and sodium chloride were purchased from Sinopharm Chemical Reagent Co., Ltd. (Beijing, China) and were all of analytical grade. Purified water was prepared by the Milli-Q^®^ Advantage A10 purification system (Sigma-Aldrich, St. Louis, MO, USA).

Diclofenac sodium as an active pharmaceutical ingredient (API) was obtained from Xi Yue Pharma Co., Ltd. (Xi’an, China). Microcrystalline Cellulose (MCC) PH-301 was obtained from Asahi-Kasei (Tokyo, Japan). Polyvinyl Pyrrolidone (PVP) K30 was obtained from BASF (Ludwigshafen, Germany). Eudragit^®^ L30D-55, RS PO, RL PO and Aerosil 200 were obtained from Evonik (Essen, Germany). Triethyl citrate (TEC) was obtained from Bengbu BBCA Tushan Pharmaceutical Co., Ltd. (Bengbu, China). Propylene glycol (PG) was obtained from Well Pharmaceutical Co., Ltd. (Nanjing, China). Talc was obtained from Longsheng Talc Development Co., Ltd. (Guilin, China).

The Beagle dogs were purchased from Shanghai Jiaotong University College of Agricultural and Biological Sciences Experimental Practice Field (Shanghai, China). The bioequivalent experiments were approved by the Ethics committee for the care and use of laboratory animals at the School of Pharmacy, Fudan University (approval number of the Ethics: 2023-05-YJ-WJX-141).

### 2.2. PBPK Modeling Approach

All data on API properties were entered in GastroPlus^TM^ as shown in Table 1. The solubility of Diclofenac sodium was conducted in the condition of 37 °C and 150 rpm for 24 h using a constant temperature shaker. Human PK data after administration of intravenous (75 mg, *n* = 18), oral enteric-coated tablets (100 mg, *n* = 12) and Difene^®^ (75 mg, *n* = 40) were obtained from the published literature. Observed mean plasma concentration profiles from the literature were acquired using Digit (Simulation Plus, Inc., version 1.04, USA). The type of compartmental models were simultaneously fitted using the PKPlus^TM^ module. The PBPK model was built using an ACAT model with a 65 kg Chinese healthy male as the virtual subject and was optimized by referring to the PK parameters of Difene^®^. The built model was evaluated by comparing the predicted and observed in vivo profiles of enteric-coated tablets. The folding error (FE) ratio of PK parameters between predicted and observed values was calculated and served as the internal validation of the model. The calculation formula for FE ratio is as follows:

FE ratio = Vp/Vo (1)

In the formula, Vp is the predicted value and Vo is the observed value, according to FDA, EMA and NMPA (China) guidelines of bioequivalence. The ratio range of PK parameters should be in the range of 0.80–1.25.

### 2.3. In Vitro Release Test of Difene^®^

#### 2.3.1. Analytical Methods for Release Tests

The samples were collected from a dissolution apparatus 708DS-850DS (Agilent Technologies Inc., Santa Clara, CA, USA) and were determined with a high-performance liquid chromatography system (Agilent 1260 Infinity II). The analytical column was ZORBAX XDB-C18 (4.6 × 150 mm, 5 μm) from Agilent Technologies Inc. (USA). The mobile phase was a mixture of methanol and 4% acetic acid solution (70:30) at a flow rate of 1.0 mL/min; the oven temperature was 30 °C; the injection volume was 20 μL; and the detection wavelength was set at 281 nm.

#### 2.3.2. Media Conditions of Release Tests

The contents of the capsule were tested using a basket apparatus (USP apparatus 1, rotational speed of 100 rpm). The volume of the vessels was 1000 mL and the temperature was maintained at 37 °C ± 0.5 °C throughout each release run. Six different media conditions were prepared and used in release tests as shown in Table 2. Approximately 5 mL samples were withdrawn and filtered through a 0.45 μm membrane immediately. The samples were analyzed using the HPLC method described in Section 2.3.1. All experiments were conducted in six parallel.

### 2.4. IVIVR of Difene^®^ in Different Release Conditions

The release profiles of Difene^®^ in different media conditions were input and single, double and triple Weibull functions were fitted using the Weibull function module. The Weibull function is as follows:

(2) $$\%DoseReleased=Max\times (1-f_{1}exp\left[\frac{{-(t-T)}^{b_{1}}}{A_{1}}\right]{-f}_{2}exp\left[\frac{{-(t-T)}^{b_{2}}}{A_{2}}\right]{-f}_{3}exp\left[\frac{{-(t-T)}^{b_{3}}}{A_{3}}\right])$$

where *Max* is the total released dose, f is fraction, *A* is time scale and *b* is the shape parameters. The best fitting function was selected according to the value of correlation coefficient (R) and AIC. The optimized control release profiles and parameters were saved as in vitro data for IVIVR simulation.

The observed mean plasma concentration profile of Difene^®^ from the literature was acquired using Digit and was saved as in vivo data. IVIVR simulations were conducted using the built PBPK model with a 65 kg Chinese healthy male as the virtual subject. The FE ratio of PK parameters between predicted and observed values was calculated and served as the validation of IVIVR.

### 2.5. Preparation of Generic Capsules of Diclofenac Sodium

The generic dual-release enteric-coated capsules were prepared by coating with functional polymer materials using a fluid-bed coater equipped with a Wurster column (FLZB-1.5, Chanse Technology Inc., Changzhou, China). The drug-loaded pellets were prepared by a three-step process involving wet granulation, extrusion–spheronization and fluid-bed drying sequentially. MCC, PVP and Aerosil 200 were used as excipients in the formula of drug-loaded pellets.

The ER pellets were coated with Eudragit^®^ L30D-55 as the polymer. A 10% *w*/*w* propylene glycol based on polymer was used as a plasticizer and a 50% *w*/*w* talc based on polymer was added as an anti-tacking agent. The pH value of enteric coating dispersion was adjusted to 5.2 using 1 M sodium hydroxide. Three levels of weight gain were designed, i.e., 24%, 32% and 40%, as shown in Table 3.

The SR pellets were coated with a combination of Eudragit^®^ RS PO and RL PO as the polymer. A 10% *w*/*w* TEC based on polymer was used as a plasticizer and a 75% *w*/*w* talc based on polymer was added as an anti-tacking agent. Three levels of polymer RS-RL ratios and weight gain were designed as shown in Table 4.

Quality-related media condition was used in release tests for formulation screening of ER and SR pellets. In addition, media condition 4 in Table 2 was employed for acid resistance in the release test of ER pellets. F3 of SR in Table 4 was used as an SR pellet in enteric formulation screening. F3 of ER in Table 3 was used as an ER pellet in sustained-release formulation screening. The f2 value was calculated and assessed the similarity of release profiles of the reference and generic formulations using the standard mathematical equation as follows:

(3) f2 = 50 × log (1 + 1/n × ∑(Rt−Tt))

where n is the number of sample times and Rt and Tt are the mean percent dissolved at each time point for the reference and test medium, respectively.

Pellets of ER (with a label of 25 mg) and SR (with a label of 50 mg) were filled into a hard gelatin capsule shell, size “2” (Lonza Capsugel Ltd., Suzhou, China) after screening as the generic capsule for the bioequivalence experiment.

### 2.6. Pharmacokinetics of Bioequivalence Test in Beagle Dogs

#### 2.6.1. LC-MS/MS Method for Determination of Diclofenac Sodium in Plasma

A 6500 Qtrap^TM^ LC-MS/MS system from AB SCIEX was used for plasma analysis. The analytical column was an Eclipse Plus C18 RRHD (2.1 × 50 mm, 1.8 μm) from Agilent Technologies Inc. (USA). The mobile phase was a mixture of 0.1% formic acid solution and acetonitrile (20:80) at a flow rate of 0.3 mL/min, the oven and auto-sampler temperature were 40 °C and 15 °C, respectively. The injection volume was 1 μL. The MS with electron spray ionization (ESI) source in mode of multiple reaction monitoring (MRM) was used for analysis. The temperature of the vaporizer was set at 500 °C. The pressure of CUR, GS1 and GS2 was set at 35 psi, 50 psi and 50 psi, respectively. Negative ion mode was used for Diclofenac sodium and positive ion mode was used for IS. Collision energy was −15 V for Diclofenac sodium and 28 V for IS. Quantification was performed using MRM of the transition ions m/z 294.0→250.2 and m/z 358.2→139.0 for Diclofenac sodium and IS, respectively.

#### 2.6.2. Preparation of Standard Plasma Solutions

The stock solutions of Diclofenac sodium (80 μg/mL) and IS (160 ng/mL) were prepared by dissolving and diluting with acetonitrile. Then, a series of gradient concentrations (0.08–40 μg/mL) of standard solutions were diluted with acetonitrile. To prepare the standard plasma solution, 50 μL of standard solutions, 250 μL of IS and 100 μL of blank plasma were added and vortexed for 1 min. The mixture was centrifuged at 12,000 rpm at 4 °C for 5 min and the supernatant was transferred into LC-MS/MS for analysis and validation.

#### 2.6.3. Pharmacokinetic Study of Bioequivalence

A single Difene^®^ capsule or generic dual-release enteric-coated capsule was administered orally by the design of a randomized crossover study. Six beagle dogs were fasted for 24 h before the experiment. Foreleg venous blood samples of 2 mL were collected before and after the drug administration at 0.25, 0.5, 0.75, 1, 1.5, 2, 3, 4, 6, 8, 12 and 24 h. The blood samples were prepared by centrifugation at 5000 rpm for 10 min. The obtained plasma samples were stored at −80 °C until analysis.

#### 2.6.4. Pharmacokinetic Data Analysis

PK profiles of the Brand Name and generic capsule were plotted. AUC_0–t_ and AUC_0–∞_ were calculated by the trapezoidal rule. All the pharmacokinetic and bioequivalence parameters were calculated by DAS software (version 2.0).

## 3. Results

### 3.1. PBPK Model for Difene^®^

The foundational pharmacokinetic parameters were determined from mean plasma concentration profiles of intravenous and oral enteric-coated tablets. One- to three-compartment models were fitted from the profile of oral enteric-coated tablets using the PKPlus^TM^ module. The best-fitting compartment and values of correlation coefficients, Akaike information criterion (AIC) and Schwarz Criterion (SC) are shown in Table 5. The AIC and SC values of three compartmental models differed minimally, while the maximum correlation coefficient of a model was the two-compartment with a value of 0.921.

**Figure 1:**
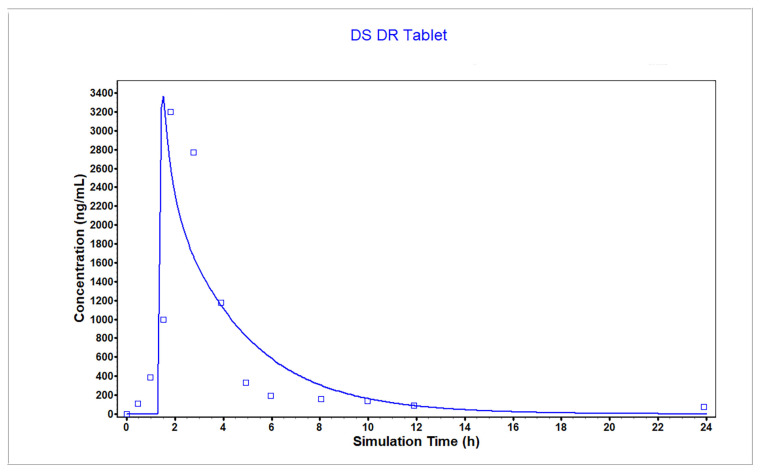
Fitting effect of observed and simulated concentration–time curve after oral administration of enteric-coated tablet.

The plasma concentration profile and PK parameters of the enteric-coated tablet were simulated and compared to the observed values from the literature as evaluation of the model, which is shown in Figure 1. The results of FE ratio of C_max_, AUC and T_max_ between predicted and observed models presented in Table 6 are 1.05, 0.93 and 0.84, respectively.

**Figure 2:**
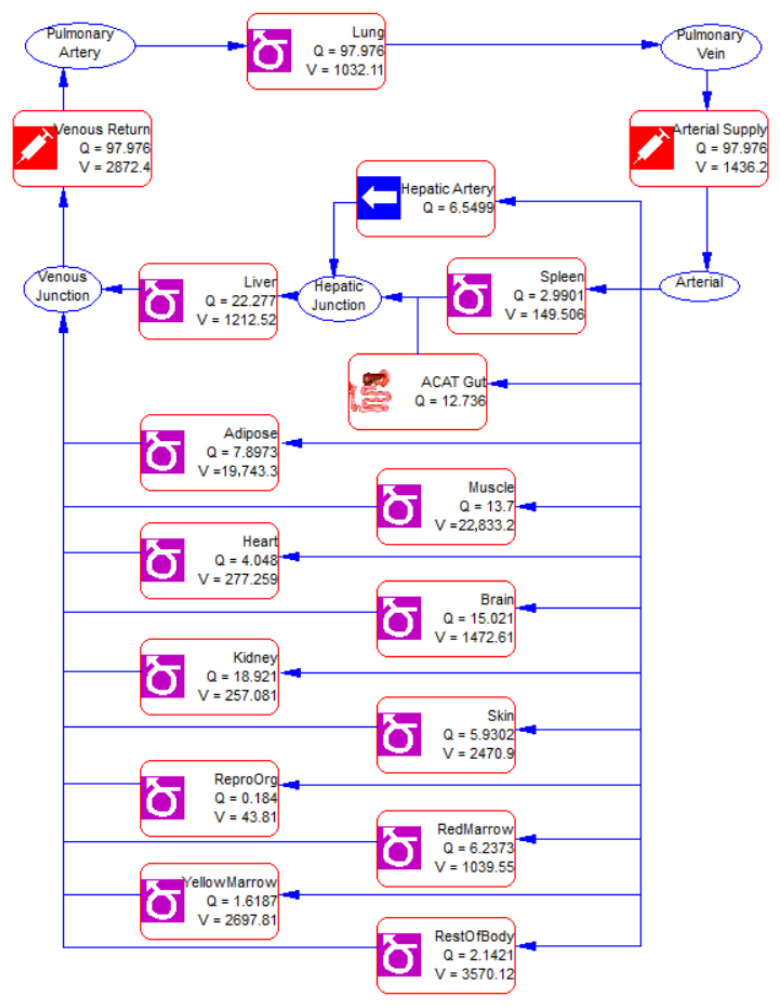
Recurrent blood flow diagram of Diclofenac sodium.

With the foundational model, the PBPK model for Difene^®^ was optimized using the ACAT model and referring the PK parameters of the reference from the literature. Clearance of the reference was fixed at 18.06 L·h^−1^. Recurrent blood flow diagram of Diclofenac sodium was built, which is shown in Figure 2. Parameters in major tissues and organs were calculated using the Lukacova (Rodegers–Single) formulation which is presented in Table 7.

### 3.2. In Vitro Release of Diclofenac Sodium from Difene^®^

**Figure 3:**
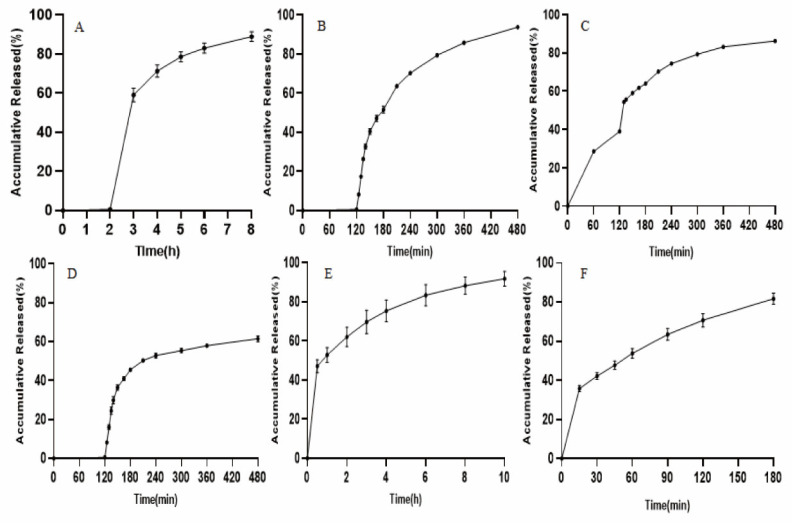
Release profiles of Diclofenac sodium from Difene^®^ in pH 1.2–pH 6.8 (**A**), pH 1.2–pH 6.0–pH 6.8 (**B**), pH 4.0–pH 6.0–pH 6.8 (**C**), pH 1.2–pH 6.0 (**D**), pH 6.8 (**E**), and water (**F**) (*n* = 6).

The results of release tests of Brand Name in different media conditions are shown in Figure 3. In this study, the requirements of the leakage condition in final media were met. The release profiles of six media conditions were further used as in vitro data for IVIVR simulation.

### 3.3. IVIVR Developed for Difene^®^

**Figure 4:**
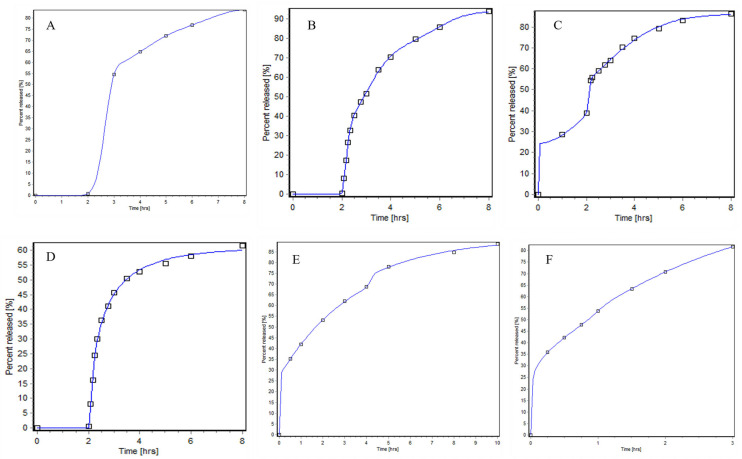
Weibull function profiles in pH 1.2–pH 6.8 (**A**), pH 1.2–pH 6.0–pH 6.8 (**B**), pH 4.0–pH 6.0–pH 6.8 (**C**), pH 1.2–pH 6.0 (**D**), pH 6.8 (**E**), and water (**F**).

Before IVIVR simulation, the type of Weibull function for six mediums were evaluated and Weibull parameters were fitted. The fitting Weibull function and evaluation metrics are presented in Table 8, the parameters of the Weibull function formulation are presented in Table 9, profiles are shown as Figure 4. As shown in Table 8, the best-fitting Weibull function for all media was the triple Weibull function, with the correlation coefficients of 1.0000, 0.9998, 0.9993, 0.9999, 0.9995 and 1.0000, respectively. The correlation coefficients of double and triple Weibull for pH 1.2-pH 6.8 media was identical; however, the values of AIC and SC for triple were lower than double Weibull.

**Figure 5:**
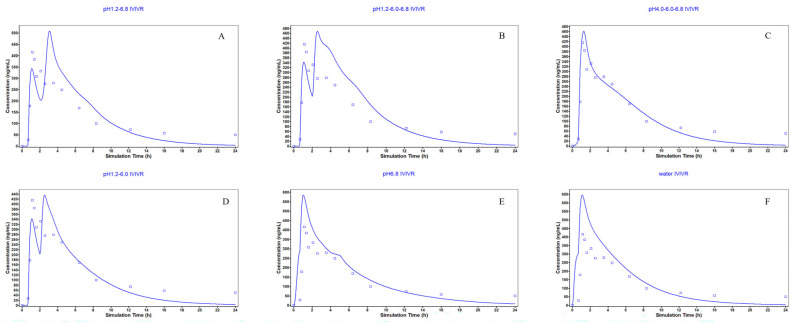
Validation of IVIVR in pH 1.2–pH 6.8 (**A**), pH 1.2–pH 6.0–pH 6.8 (**B**), pH 4.0–pH 6.0–pH 6.8 (**C**), pH 1.2–pH 6.0 (**D**), pH 6.8 (**E**) and water (**F**).

Observed mean plasma concentration profile of Difene^®^ from the literature was used as in vivo data. IVIVR simulations of six media were developed using in vitro and in vivo data with the built PBPK model. The FE ratio of C_max_, AUC and T_max_ between predicted and observed values is calculated and is shown in Table 10; profiles are shown in Figure 5. The results show that the FE ratios of pH 4.0–pH 6.0–pH 6.8 were all in a range of 0.80–1.25, with values of 1.11, 0.86 and 1.11. The FE ratio of either AUC or C_max_ for the other media was out of limitation. Therefore, according to the validation of IVIVR, pH 4.0–pH 6.0–pH 6.8 media showed the best relationship of in vitro and in vivo of Diclofenac sodium dual-release enteric capsule in six media and was selected as the quality-related media condition for formulation screening of generic capsules.

### 3.4. Formulation Screening of Generic Capsule

#### 3.4.1. Release Test of Different Weight Gains of Eudragit^®^ L30D-55

**Figure 6:**
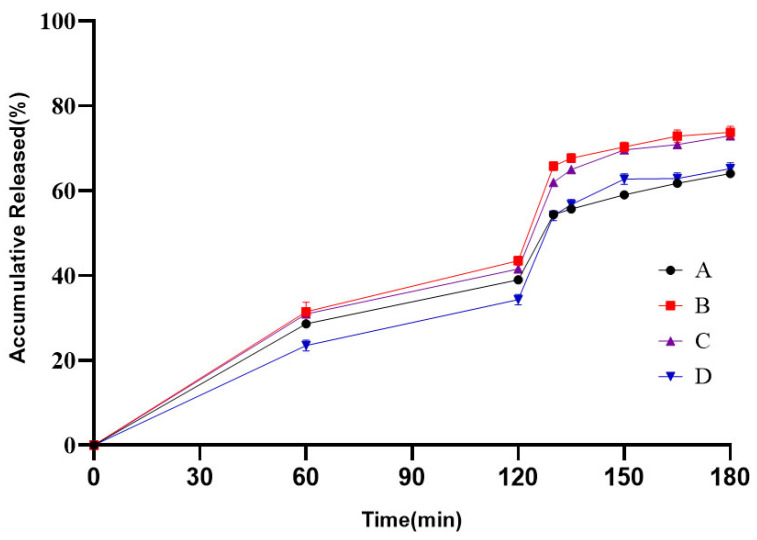
Release profiles of the reference (A), ER F1 (B), ER F2 (C) and ER F3 (D) in pH4.0–pH6.0 stage of the quality-related media (*n* = 6).

Different levels of L30D-55 weight gains were designed at 15%, 20%, and 25%, as ER F1, F2 and F3 presented in Table 3. Due to rapid dissolution of L30D-55 in media above pH 5.5, the weight gain screening for the ER pellets of the generic product was conducted primarily under the pH 4.0–pH 6.0 stage of the quality-related media condition. The weight gains of the ER pellets were selected by comparing the release profiles between various weight gain formulations and the reference product, which are shown in Figure 6. The results demonstrate that ER pellets prepared with a 25% L30D-55 weight gain (F3) exhibit the highest curve fitting with the reference product in the pH 4.0–pH 6.0 range, with the most favorable f2 factor calculation presented in Table 11. Although the f2 factors for the ER F1 and ER F2 formulations both met the acceptance criterion of greater than 50, the values approached the threshold closely. Furthermore, the trends of the release profile indicate that formulations of ER F1 and ER F2 exhibit faster release rates than the reference product at the pH 6.0 stage. Consequently, among the three designed formulations, the ER F3 demonstrated the best consistency with the reference product.

**Figure 7:**
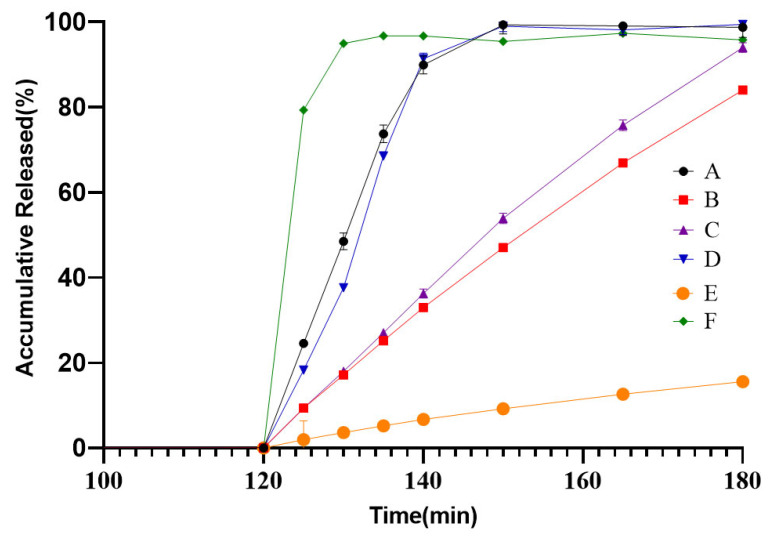
Release profiles of the reference (A), ER F1 (B), ER F2 (C), ER F3 (D), drug-loaded core pellets (E) in pH 1.2–pH 6.0 media and drug-loaded core pellets without acid treatment (F) in pH 6.0 media. (*n* = 6).

Furthermore, to verify the acid tolerance of the ER pellets, a release media (No. 4 in Table 2) of pH 1.2–pH 6.0 was employed as the test condition for evaluating both the ER pellets and the drug-loaded cores. The release profiles of different formulations are shown in Figure 7. The results indicate that the enteric-coated pellets of all three formulations demonstrated drug release not exceeding 10% after 2 h in a pH 1.2 hydrochloric acid medium, meeting the requirements of acid stability. However, the dissolution profile for the drug-loaded cores after 2 h exposure to acid medium was substantially lower compared to the cores without acid treatment. Previous studies [30] have reported that Diclofenac sodium may partially convert to its free acid form under acidic conditions, forming an insoluble film on the surface that impedes subsequent release. Examination of the release profiles of different weight gains of ER pellets demonstrate that the F1 and F2 formulations exhibited inferior acid tolerance, presumably due to acid medium penetration into the pellet cores leading to poorly soluble drug transformation that impeded API release in pH 6.0 media. In contrast, the 25% weight gain formulation displayed high curve fitting with the reference product, with an f2 factor exceeding 50 presented in Table 12.

#### 3.4.2. Release Test of Different Ratio of RS PO and RL PO

**Figure 8:**
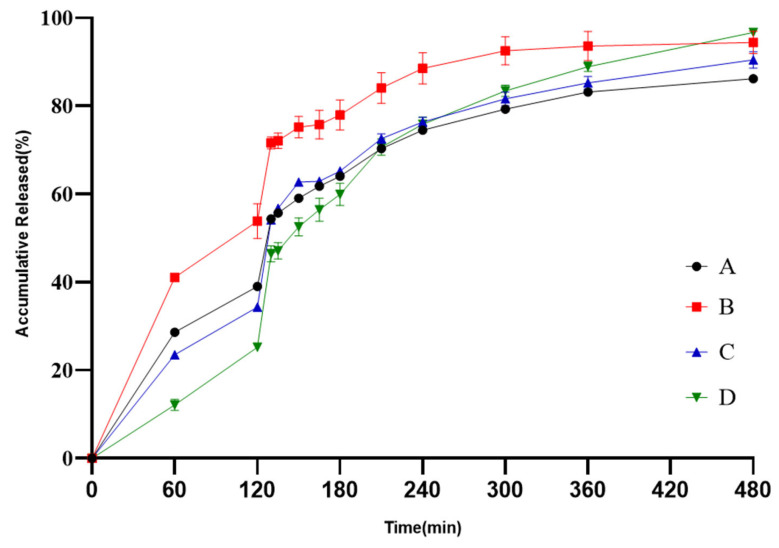
Release profiles of the reference (A), SR F2 (B), SR F3 (C) and SR F4 (D) in quality-related media (*n* = 6).

Different levels of ratio of RS PO and RL PO were designed at 2:1, 3:1, and 4:1, as SR F2, F3 and F4 presented in Table 4. The ratio of two polymers in SR pellets was selected by comparing the release profiles between various weight gain formulations and the reference product in a quality-related condition, which are shown in Figure 8. The results demonstrate that SR pellets prepared in the SR F3 formulation exhibited the highest curve fitting with the reference product, with the most favorable f2 factor calculation as presented in Table 13. Although the f2 factors for the F4 formulation met the acceptance criterion of exceeding 50, the value approached the threshold closely. Furthermore, the trends of the release profile indicate that formulations of F3 exhibited slower release rates than the reference product in quality-related media. Consequently, among the three designed formulations, a ratio of RS PO and RL PO at 3:1 was selected as the polymer proportion for the sustained-release pellets.

#### 3.4.3. Release Test of Different Weight Gains of SR Pellets

**Figure 9:**
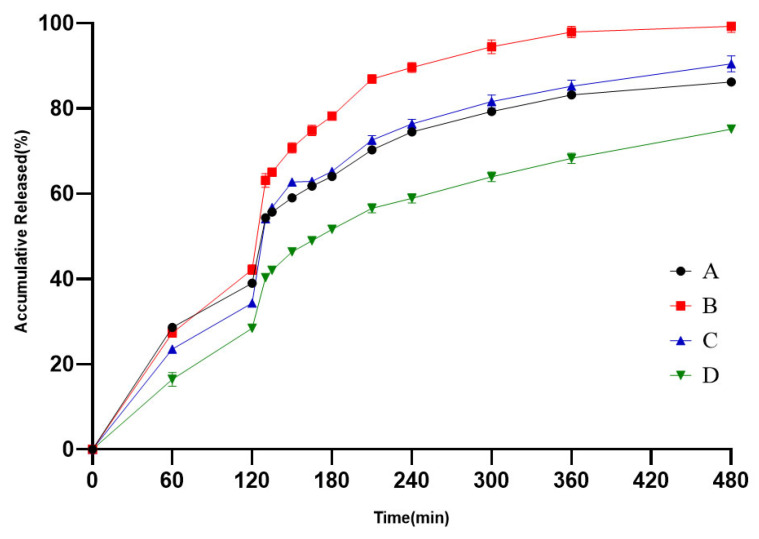
Release profiles of the reference (A), SR F1 (B), SR F3 (C) and SR F5 (D) in quality-related media (*n* = 6).

Different levels of weight gains of SR pellets were designed at 5.9%, 8.9%, and 11.9%, as SR F1, F3 and F5 presented in Table 4. The weight gains of the SR pellets were selected by comparing the release profiles between the designed formulations and the reference product in the quality-related condition, which are shown in Figure 9. The results demonstrate that the release rate of the SR pellets decreased with increasing weight gains. Among the three designed formulations evaluated, the formulation of SR F3 exhibited the highest curve fitting with the reference product in the quality-related media, with the most favorable f2 factor. In comparison, the f2 factors for SR F1 and SR F5 failed to meet the acceptance criterion, and are presented in Table 14. Consequently, a coating weight gain of 8.9% was selected for the sustained-release pellets.

### 3.5. LC-MS/MS Method Validation of Diclofenac Sodium in Plasma

**Figure 10:**
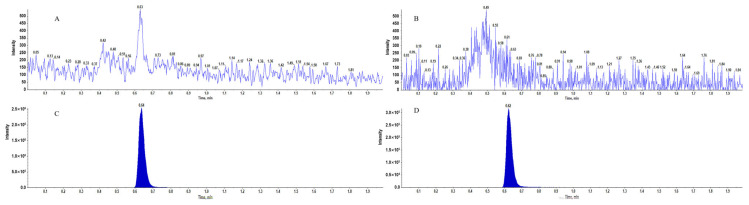
LC-MS/MS chromatogram of blank plasma (**A**—Diclofenac sodium, **B**—IS) and plasma with standard (**C**—Diclofenac Sodium, **D**—IS).

The LC-MS/MS chromatograms of Diclofenac sodium and IS in blank and plasma are shown in Figure 10, which indicates a good selectivity of the method. The standard curve of Diclofenac sodium, obtained by calculating the peak areas ratio of standard and IS at different concentrations, as shown in Table S1 and Figure S1, indicates a good correlation in the range of the method. The recoveries of standard in different concentrations ranged from 97.88% to 106.69%, and the intra- and inter-day precisions ranged from 1.78% to 4.74% and 2.42% to 6.52%, respectively, with detailed results in Tables S2 and S3. The stability tests indicate that Diclofenac sodium could be stable for 24 h at room temperature and 4 °C and three freeze–thaw cycles, with detailed results in Table S4.

### 3.6. Bioequivalence of Generic Product and Reference in Beagle Dogs

**Figure 11:**
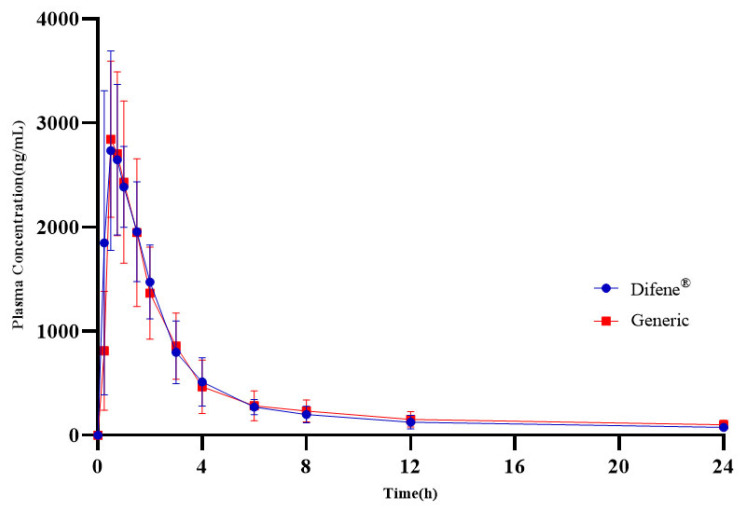
PK profiles in beagle dogs after administration of the reference and the generic formulation (*n* = 6).

As shown in Figure 11, the plasma concentration–time curves of Diclofenac sodium of the generic product and the reference in beagle dogs were developed after oral administration. The mean pharmacokinetics parameters and the statistical analysis of bioequivalence of the generic and the reference product are presented in Table 15 and Table 16, respectively. The results of statistical analysis indicate that the geometric mean ratio (GMR) for AUC_(0→t)_, AUC_(0→∞)_ and C_max_ was 102.2%, 105.2% and 97.7%, respectively. Meanwhile, the 90% confidence interval of the GMR for AUC_(0→t)_, AUC_(0→∞)_ and C_max_ was 90.0% to 112.8%, 90.0% to 121.4% and 87.9% to 106.8%, respectively. The results of GMR and 90% confidence intervals both met the bioequivalent criterion of 80.0% to 125.0%. Nonparametric analysis of T_max_ was conducted using the Wilcoxon signed-rank test for comparisons, which is displayed in Table 17. The results demonstrate that the nonparametric analysis of T_max_ yielded a *p*-value of 0.858, indicating no statistically significant difference in T_max_ between the generic product and the reference.

## 4. Discussion

Dual-release sustained formulations combine the advantages of rapid drug release with prolonged maintenance of therapeutic plasma concentrations. However, the integration of two distinct release mechanisms substantially increases the complexity of generic development. The conventional trial-and-error approach requires comparison of curve fitting between generic and reference products in multiple media conditions to assess the risk of the bioequivalence study. This method, with substantial workload and poor specificity, is not suitable for generic development of complex dosage forms such as dual-release sustained formulations. Accordingly, we sought to establish a workflow wherein PBPK models and IVIVR are pre-established to identify quality-related media conditions for the reference product to improve the efficiency and the success rate of generic development for dual-release sustained products.

GastroPlus™ is a simulation platform and a modeling tool commonly used in pharmaceutical development and pharmacokinetic research. It can construct PBPK base models through incorporation of intravenous and IR or ER pharmacokinetic data, obtained from experimental or published sources. An ACAT model is usually employed as the absorption model; parameters including drug physicochemical properties, gastrointestinal pH environments, and gastric emptying times are integrated for evaluation of variations in gastrointestinal absorption of orally administered drugs and simulation of in vivo absorption and bioequivalence for generic products. In addition, the software also enables establishment of linear or nonlinear relationships between in vitro release and in vivo absorption through PBPK modeling, thereby determining quality-related media for formulation development and screening. Similar investigations [31,32] have been reported in the literature, providing a theoretical reference for the present study.

A traditional IVIVC (in vitro and in vivo correlation) model describes a liner mathematical relationship between in vitro dissolution properties and in vivo pharmacokinetics of formulations. According to the FDA guidelines, IVIVC can be divided into Level A, B, and C. Generally, IVIVC Level A is regarded as the widest regulatory acceptance, requiring point-to-point correlation and both internal and external validation by using different formulation release-rate profiles. In contrast to IVIVC, IVIVR is a nonlinear mathematical model which extends the concept of IVIVC [33]. Strictly speaking, it does not fall into any IVIVC category and has no official classification now. Instead of establishing a traditional point-to-point correlation, the objective of our study is to develop a mechanistically informed in vitro–in vivo linkage using PBPK modeling. In this context, we constructed an integrated PBPK–IVIVR-based framework to support the identification of quality-related media and to guide formulation screening for dual-release sustained formulations, thereby quantitatively linking in vitro release behavior with in vivo pharmacokinetic performance.

We selected Diclofenac sodium as the model drug for the following reasons. First, Difene^®^ is a well-established commercial product widely used in clinical practice, and no generic product has been approved for marketing in China. Second, as a BCS Class II compound, the dissolution behavior of API directly influences the extent of its absorption in vivo, making the development of an IVIVR for the reference product particularly critical. While published studies have described the use of PBPK modeling and IVIVR for enteric-coated formulations of Diclofenac sodium to assess formulation performance and predict in vivo absorption [34,35], no such work has been reported specifically for the dual-release enteric-coated capsule. For this reason, we selected Difene^®^ as the reference product to explore a generic development strategy for dual-release sustained formulations.

In this study, published pharmacokinetic data from oral enteric-coated tablets and Difene^®^ administered to Chinese subjects were employed for PBPK model construction, with the aim of improving its specificity to the target population. A 65 kg Chinese male was selected as the physiological model within the software to closely match the demographic characteristics of the source data. By aligning the physiological model parameters (e.g., body weight, sex, and ethnicity) with the reported study population, we aimed to minimize potential variability arising from population mismatch and to improve the internal consistency of the model.

Although use of a single “representative” virtual subject may not fully capture inter-individual variability, given that the source pharmacokinetic data were reported as mean profiles from a relatively homogeneous population, we consider this approach appropriate for the purpose of model development and IVIVR establishment. Moreover, the satisfactory agreement between predicted and observed pharmacokinetic parameters, with fold error values within 0.80–1.25, further supports that potential residual variability did not significantly impact model performance in this study.

Beside the pharmacopeia, drug physicochemical properties and physiological conditions were also taken into consideration in the design of the release media in the present study. The pH4.0–pH6.0–pH6.8 media was designed based on following considerations. First, the saturation solubility experiments demonstrated that Diclofenac sodium exhibited a pH-dependent solubility characteristic, with solubility increasing as pH rises. Selection of a lower pH value as the initial stage of media might fail to reflect the minor release from sustained-release pellets accurately. Second, while the pH value of the normal human stomach ranges from 1.35 to 3.5 [36], the critical release and absorption processes for this formulation primarily occur after gastric emptying, in the intestinal environment. This is also supported by pharmacokinetic data from the literature, which indicate evident absorption of the reference product within the first hour. Therefore, we consider a sub-acidic condition such as pH 4.0 in the initial stage is more relevant for capturing the onset of drug release. Meanwhile, media at pH 6.0 and 6.8 are widely employed to simulate the pH environment of the human intestine. We consider this pH combination to better simulate the environmental conditions for in vivo release and absorption of this formulation. In addition, a dissolution database from FDA of similar products [37] also provided a supporting rationale for this media design.

The results of IVIVR for the reference product demonstrated that the pH4.0–pH6.0–pH6.8 media exhibited the optimal in vitro–in vivo relationship, with FE ratios between predicted and observed values for AUC, C_max_, and T_max_ all falling within acceptable ranges. This media was consequently adopted as the quality-related media for critical formulation parameters screening (e.g., coating weight gain, polymer ratio, etc.) of the generic product.

Additionally, due to the requirement of the acid resistance test for enteric-coated pellets in pharmacopeia, a pH 1.2–pH 6.0 media was employed as a secondary screening condition for ER pellets development. The optimized formulation for ER and SR pellets of the generic product were established through this approach.

To evaluate preliminary bioequivalence between the generic and reference products and formulation development framework, beagle dogs were employed as the model species and pharmacokinetic studies were conducted using a two-period crossover design. Results demonstrated that the generic product, developed with quality-related media as the primary screening criterion, exhibited no statistically significant differences from the reference product in beagle dogs for major pharmacokinetic parameters including AUC, C_max_, and T_max_, with geometric mean ratios and 90% CI for these parameters within bioequivalence acceptance criteria.

Compared with the conventional approach of generic development, this study employed a pre-established PBPK model and IVIVR investigation of the reference product to screen quality-related media conditions. This approach narrowed the scope of screening parameters, improved the specificity of the development process, and reduced the overall workload. The bioequivalence outcome of the pharmacokinetic study in beagle dogs confirmed the validity of using a PBPK model to identify quality-related media for guiding the generic development of dual-release sustained formulations. The strategy described here may serve as a reference for the generic development of other dual-release sustained products.

## 5. Conclusions

In this study, Difene^®^ was selected as the reference product. The PBPK model for the reference product was constructed using GastroPlus^TM^ as the modeling tool, and IVIVR was established based upon in vitro release profiles to determine the quality-related media condition of the reference. Employing the quality-related media as the screening condition, the coating weight gains and polymer ratios for both enteric-coated and sustained-release pellets of the generic product were evaluated, and the final formulation of the generic product was established. Pharmacokinetic studies in beagle dogs demonstrated that the developed generic product was bioequivalent to the reference product in vivo. Overall, this approach minimized interference from non-related media during formulation development and enhanced the targeting of formulation screening through establishing the IVIVR and identifying a quality-related condition based on the PBPK model before formulation development, and provided a reliable strategy for the generic development of other dual-release sustained formulations.

## 6. Limitations

In the present study, the formulation with the highest f2 factor in quality-related media was selected as the optimal candidate and was subsequently demonstrated to be bioequivalent to the reference product in vivo. This provides indirect but meaningful evidence that the selected media is capable of capturing critical formulation attributes relevant to in vivo performance. However, the absence of a negative control formulation (i.e., one with low similar values in vitro) limits the ability to fully evaluate the discriminatory power of the media across a broader range of formulation differences. At the current stage, such investigations are limited by the lack of human PK data for the generic product. In future work, parameter sensitivity analysis (PSA) and population virtual bioequivalence assessment of formulations with low similarity factor values could be conducted using the PBPK model, after pharmacokinetic data of the generic product obtained from pilot bioequivalence studies in humans, to establish acceptable ranges for in vitro similarity factors. In addition, the risk of bioequivalence failure for the generic product could be predicted through population-based virtual bioequivalence studies.

## Figures and Tables

**Table 1:** API properties used for modeling.

Parameter	Value	Reference
Molecular weight (g/mol)	296.149	Data base [26]
log*P*	4.51	
pKa	4 (Acid); −2.1 (Base)	
Dose and form (mg)	75 (I.V.: Infusion)	Literature [27]
	100 (DR: Tablet)	Literature [28]
	75 (Mixed Multiple Dose)	Label
Dose volume (mL)	250	Default
Solubility (mg/mL)	1.69 × 10^−3^@pH1.2	Tested
	1.35 × 10^−2^@pH4.0	
	1.64 × 10^−2^@pH4.5	
	2.46@pH6.0	
	9.63@pH6.8	
Mean precipitation time (s)	900	Default
Drug particle density (g/mL)	1.2	Default
Diffusion coefficient (cm^2^/s × 10^5^)	0.75	Default
Peff (×10^−4^ cm/s)	7.79	Literature [29]

**Table 2:** Media conditions of release tests.

No.	Media	Sampling Times
1	2 Stage release: 0.1 M HCl at pH1.2 (0–2 h), transferred to 0.05 M phosphate and 0.1 M sodium chloride buffer at pH6.8 (2–8 h).	2, 3, 4, 5, 6 and 8 h
2	3 Stage release: 0.1 M HCl at pH1.2 (0–2 h), transferred to 0.05 M phosphate and 0.1 M sodium chloride buffer at pH6.0 (2–3 h) and 6.8 (3–8 h).	120, 125, 130, 140, 150, 165, 180, 210, 240, 300, 360 and 480 min
3	3 Stage release: 0.05 M phosphate and 0.1 M sodium chloride buffer at pH4.0 (0–2 h), 6.0 (2–3 h) and 6.8 (3–8 h).	60, 120, 125, 130, 135, 150, 165, 180, 210, 240, 300, 360 and 480 min
4	2 Stage release: 0.1 M HCl at pH1.2 (0–2 h), transferred to 0.05 M phosphate and 0.1 M sodium chloride buffer at pH6.0 (2–8 h).	120, 125, 130, 135, 140, 150, 165, 180, 210, 240, 300, 360 and 480 min
5	0.05 M phosphate and 0.1 M sodium chloride buffer at pH6.8 (10 h)	0.5, 1, 2, 3, 4, 6, 8 and 10 h
6	Water (3 h)	15, 30, 45, 60, 90, 120 and 180 min

**Table 3:** Formulation design of ER pellets.

	ER F1	ER F2	ER F3
L30D-55	15.0%	20.0%	25.0%
PG	1.5%	2.0%	2.5%
Talc	7.5%	10.0%	12.5%
Weight gain	24.0%	32.0%	40.0%

**Table 4:** Formulation design of SR pellets.

	SR F1	SR F2	SR F3	SR F4	SR F5
RS PO	2.40%	3.20%	3.60%	3.84%	4.80%
RL PO	0.80%	1.60%	1.20%	0.96%	1.60%
Talc	2.40%	3.60%	3.60%	3.60%	4.80%
TEC	0.30%	0.50%	0.50%	0.50%	0.70%
Weight gain	5.90%	8.90%	8.90%	8.90%	11.90%

**Table 5:** Values of correlation coefficients, AIC and SC for compartment evaluation.

Compartment	Weighting	R	AIC	SC
One	1/Yhat^2^	0.722	−26.97	−24.84
Two	1/Yhat^2^	0.921	−29.27	−25.73
Three	1/Yhat^2^	0.823	−39.87	−35.92

**Table 6:** Fitting effect of observed and simulated PK parameters after oral administration of enteric-coated tablet.

	Observed	Predicted	FE Ratio
C_max_	3200.0	3363.1	1.05
AUC	9166.0	8493.3	0.93
T_max_	1.81	1.52	0.84

**Table 7:** Parameters values in major tissues or organs.

Tissue/Organ	K_p_	f_ut_
Lung	0.55	0.656
Adipose	0.22	0.194
Liver	0.85	0.421
Spleen	0.17	0.254
Heart	0.22	0.199
Kidney	0.73	0.490

**Table 8:** Weibull function and coefficient parameters of six media conditions.

Media	Weibull	R^2^	AIC	SC
pH 1.2–pH 6.8	Single Weibull	0.9996	−1.1437	−1.3600
	Double Weibull	1.0000	−15.046	−15.425
	Triple Weibull	1.0000	−124.27	−124.81
pH 1.2–pH 6.0–pH 6.8	Single Weibull	0.9978	15.427	17.983
	Double Weibull	0.9991	9.5655	14.039
	Triple Weibull	0.9998	−9.6641	−3.2735
pH 4.0–pH 6.0–pH 6.8	Single Weibull	0.9643	36.659	38.918
	Double Weibull	0.9916	23.863	27.818
	Triple Weibull	0.9993	−1.6729	3.9765
pH 1.2–pH 6.0	Single Weibull	0.9970	8.0811	10.637
	Double Weibull	0.9988	1.1204	5.5938
	Triple Weibull	0.9999	−21.091	−15.570
pH 6.8	Single Weibull	0.9827	22.813	23.602
	Double Weibull	0.9974	11.660	13.041
	Triple Weibull	0.9995	3.0737	5.0459
water	Single Weibull	0.9723	21.681	21.999
	Double Weibull	0.9998	−13.044	−12.488
	Triple Weibull	1.0000	−68.830	−68.036

**Table 9:** Parameters of Weibull function formulation of six media conditions.

Media	f_1_	A_1_	b_1_	f_2_	A_2_	b_2_	f_3_	A_3_	b_3_
pH 1.2–pH 6.8	0.6696	2.5817	4.8245	0.1200	23,110	6.0305	0.2103	27.834	3.1382
pH 1.2–pH 6.0–pH 6.8	0.4121	0.1194	1.5809	0.3784	2.1731	2.1187	0.2096	204.95	3.7696
pH 4.0–pH 6.0–pH 6.8	0.5482	10.287	1.8925	0.1731	3.18 × 10^15^	47.512	0.2787	0.0185	0.9199
pH 1.2–pH 6.0	0.5013	0.0934	1.6689	0.3629	1.0172	1.7089	0.1358	209.63	3.7404
pH 6.8	0.6436	3.4035	1.0107	0.0549	6.59 × 10^22^	0.2476	0.3015	0.2476	6.727 × 10^−4^
water	0.5659	3.1599	1.2309	0.0237	1.2226	9.2355	0.4104	0.4266	0.2812

**Table 10:** FE ratio of PK parameters between observed and predicted values of six media conditions.

Media	Observed			Predicted			FE ratio		
	C_max_	AUC	T_max_	C_max_	AUC	T_max_	C_max_	AUC	T_max_
pH 1.2–pH 6.8	416.0	2780.2	1.15	508.3	2800.0	3.04	1.22	1.01	2.64
pH 1.2–pH 6.0–pH 6.8	416.0	2780.2	1.15	468.8	2993.6	2.56	1.12	1.08	2.23
pH 4.0–pH 6.0–pH 6.8	416.0	2780.2	1.15	462.0	2387.9	1.28	1.11	0.86	1.11
pH 1.2–pH 6.0	416.0	2780.2	1.15	435.0	2384.6	2.56	1.05	0.86	2.23
pH 6.8	416.0	2780.2	1.15	585.3	3027.2	1.04	1.41	1.09	0.90
water	416.0	2780.2	1.15	654.9	3121.2	1.12	1.57	1.12	0.97

**Table 11:** Comparison of similarity factors of different enteric weight gain formulations.

Level	ER F1	ER F2	ER F3
f2 factor	50	53	75

**Table 12:** Comparison of similarity factors of enteric pellets in pH1.2–pH6.0 media.

Level	ER F1	ER F2	ER F3
f2 factor	22	23	61

**Table 13:** Comparison of similarity factors of different ratio of RS and RL formulations.

Level	SR F2	SR F3	SR F4
f2 factor	43	76	53

**Table 14:** Comparison of similarity factors of different sustained weight gain formulations.

Level	SR F1	SR F3	SR F5
f2 factor	45	76	43

**Table 15:** PK parameters of the reference and the generic formulation (*n* = 6).

	Reference	Generic
AUC_(0→t)_/ng·mL^−1^·h	8952.667 ± 2261.122	9149.841 ± 2963.332
AUC_(0→∞)_/ng·mL^−1^·h	9518.814 ± 2491.724	10,011.095 ± 3062.405
C_max_/ng·mL^−1^	3041.971 ± 717.389	2972.704 ± 844.658
T_max_/h	0.542 ± 0.292	0.542 ± 0.102
MRT/h	4.686 ± 0.730	5.292 ± 0.893

**Table 16:** Statistical analysis of AUC and C_max._

	GMR	90%CI	Criterion
AUC_(0→t)_	102.2%	90.0%~112.8%	80.0%~125.0%
AUC_(0→∞)_	105.2%	90.0%~121.4%	
C_max_	97.7%	87.9%~106.8%	

**Table 17:** Non-parametric results of T_max_ for the reference and the generic formulation.

Rank-Sum of Reference	Rank-Sum of Test	U-Value	*p*-Value
37.5	40.5	0.179	0.858

## Data Availability

The original contributions presented in this study are included in the article/Supplementary Material. Further inquiries can be directed to the corresponding author.

## Supplementary Materials

The following supporting information can be downloaded at: https://www.mdpi.com/article/10.3390/pharmaceutics18050613/s1, Figure S1: Calibration curve of Diclofenac sodium in plasma; Table S1: Calibration formulation and liner rangers of Diclofenac sodium in plasma; Table S2: Recovery of Diclofenac sodium and matrix effect in plasma; Table S3: Precision and accuracy of Diclofenac sodium in plasma; Table S4: Stability of Diclofenac sodium in plasma.

## Abbreviations

The following abbreviations are used in this manuscript:

ACAT	advanced compartmental absorption and transit
AIC	Akaike Information Criterion
API	Active pharmaceutical ingredient
ER	enteric-release
IVIVC	in vitro and in vivo correlation
IVIVR	in vitro and in vivo relationship
IS	internal standard
PBPK	Physiologically based pharmacokinetic
PK	Pharmacokinetic
PG	Propylene glycol
SC	Schwarz Criterion
SR	sustained-release
TEC	Triethyl citrate

## References

[1] Zhang Y., Fan S., Fan R., Chen Y. (2014). Research progress on dual release drug delivery systems. China Pharm..

[2] Yang M., Fan Y., Gao C. (2013). Advance on the Biphasic Drug Release System Based on Controlled Release Technology. China Pharm. J..

[3] Davanço M., Campos D., Carvalho P. (2020). In vitro—In vivo correlation in the development of oral drug formulation: A screenshot of the last two decades. Int. J. Pharm..

[4] Zhang X., Wen H., Fan J., Vince B., Li T., Gao W., Kinjo M., Brown J., Sun W., Jiang W. (2017). Integrating In Vitro, Modeling, and In Vivo Approaches to Investigate Warfarin Bioequivalence. CPT Pharmacomet. Syst. Pharmacol..

[5] Subhani S., Kim C., Muniz P., Rodriguez M., van Os S., Suarez E., Cristofoletti R., Schmidt S., Vozmediano V. (2022). Application of physiologically based absorption and pharmacokinetic modeling in the development process of oral modified release generic products. Eur. J. Pharm. Biopharm..

[6] Zhang C., Arnold S. (2025). Potential and challenges in application of physiologically based pharmacokinetic modeling in predicting diarrheal disease impact on oral drug pharmacokinetics. Drug Metab. Dispos..

[7] Chou P., Shannar A., Pan Y., Dave P., Xu J., Kong A. (2025). Application of Physiologically-Based Pharmacokinetic (PBPK) Model in Drug Development and in Dietary Phytochemicals. Curr. Pharmacol. Rep..

[8] Pang K., Durk M. (2010). Physiologically-based pharmacokinetic modeling for absorption, transport, metabolism and excretion. J. Pharmacokinet. Pharmacodyn..

[9] Foti R. (2025). Utility of physiologically based pharmacokinetic modeling in predicting and characterizing clinical drug interactions. Drug Metab. Dispos..

[10] Santos L., Jaiswal S., Chen K., Jones H., Templeton I. (2025). Real-world application of physiologically based pharmacokinetic models in drug discovery. Drug Metab. Dispos..

[11] Huang W., Bowman C., Yin M., Han L., Wen Y., Ahn S., Chen Y. (2025). A review of physiologically based pharmacokinetic modeling of renal drug disposition. Drug Metab. Dispos..

[12] Tanaka R., Irie K., Mizuno T. (2025). Physiologically Based Pharmacokinetic Modeling of Antibiotics in Children: Perspectives on Model-Informed Precision Dosing. Antibiotics.

[13] Hu C. (2021). Application of physiologically based pharmacokinetic models in consistencyevaluation of solid oral preparations. China J. Antibiot..

[14] Guideline Reporting Physiologically Based Pharmacokinetic Pbpk Modelling and Simulation. https://www.ema.europa.eu/en/documents/scientific-guideline/guideline-reporting-physiologically-based-pharmacokinetic-pbpk-modelling-and-simulation_en.pdf.

[15] Physiologically Based Pharmacokinetic Analyses Format and Content Guidance for Industry. https://www.fda.gov/regulatory-information/search-fda-guidance-documents/physiologically-based-pharmacokinetic-analyses-format-and-content-guidance-industry.

[16] Li L., Yang J. (2017). Application progress of physiologically—Based pharmacokinetic model in clinicaldevelopment of novel molecular entities. China J. Clin. Pharmacol..

[17] Gao G., Wei C. (2018). Applications of physiologically based pharmacokinetic modeling and simulation indrug development and regulatory science. China J. Clin. Pharmacol..

[18] Ruan H., Geng X., Situ Z., Shen Q., Ye T., Chen X., Su W. (2025). From In Vivo Predictive Dissolution to Virtual Bioequivalence: A GastroPlus^®^-Driven Framework for Generic Candesartan Cilexetil Tablets. Pharmaceuticals.

[19] Arafat M., Sarfraz M., AbuRuz S. (2021). Development and In Vitro Evaluation of Controlled Release Viagra^®^ Containing Poloxamer-188 Using Gastroplus™ PBPK Modeling Software for In Vivo Predictions and Pharmacokinetic Assessments. Pharmaceuticals.

[20] Vallejo C., Meaney C., Clemens L., Yang K., Lukacova V., Zhou H. (2025). Physiologically Based Pharmacokinetic Models for Infliximab, Ipilimumab, and Nivolumab Developed with GastroPlus to Predict Hepatic Concentrations. Pharmaceutics.

[21] Jones H.M., Parrott N., Ohlenbusch G., Lavé T. (2006). Predicting Pharmacokinetic Food Effects Using Biorelevant Solubility Media and Physiologically Based Modelling. Clin. Pharmacokinet..

[22] Honório Tda S., Pinto E.C., Rocha H.V., Esteves V.S., dos Santos T.C., Castro H.C., Rodrigues C.R., de Sousa V.P., Cabral L.M. (2013). In vitro-in vivo correlation of efavirenz tablets using GastroPlus^®^. AAPS PharmSciTech.

[23] Wang L., Chen J., Chen W., Ruan Z., Lou H., Yang D., Jiang B. (2023). In silico prediction of bioequivalence of atorvastatin tablets based on GastroPlus™ software. BMC Pharmacol. Toxicol..

[24] Wang X., Li L., Yang H., He Q., Zhu X., Wang J., Sun B., Liu P., Xiang X. (2025). Establishing Clinically Relevant Specifications for Carbamazepine Tablets Using Physiologically Based Pharmacokinetic Modeling. Aaps J..

[25] Schmitt W., Walter K., Kurth H.J. (1999). Clinical trial on the efficacy and safety of different diclofenac formulations: Multiple-unit formulations compared to enteric coated tablets in patients with activated osteoarthritis. Inflammopharmacology.

[26] Diclofenac Sodium. https://go.drugbank.com/drugs/DB00586.

[27] Guhmann M., Thommes M., Gerber F., Pöllinger N., Klein S., Breitkreutz J., Weitschies W. (2013). Design of biorelevant test setups for the prediction of diclofenac in vivo features after oral administration. Pharm. Res..

[28] Li Y., Xie D., Zhou Z., Wang G., Gao Y. (2004). Pharmacokinetics of diclofenac sodium delayed-action preparation. China J. New Drugs Clin. Rem..

[29] Fu H., Wang L., Hu Q., Zhang J. (2018). Dissolution and Permeability Behavior of Diclofenac Sodium enteric-Coated Tablets In-Vitro. China Pharm. J..

[30] Zhang L., Hu Y., Qin J., Fu J. (2023). Method for Evaluating In-Vivo and In-Vitro Correlation of Diclofenac Sodium Sustained-Release Tablets.

[31] Yang R., Lin Y., Chen K., Huang J., Yang S., Yao A., Yang X., Lei D., Xiao J., Yang G. (2024). Establishing Virtual Bioequivalence and Clinically Relevant Specifications for Omeprazole Enteric-Coated Capsules by Incorporating Dissolution Data in PBPK Modeling. Aaps J..

[32] Jereb R., Opara J., Legen I., Petek B., Grabnar-Peklar D. (2019). In vitro-In vivo Relationship and Bioequivalence Prediction for Modified-Release Capsules Based on a PBPK Absorption Model. AAPS PharmSciTech.

[33] Nguyen M.A., Flanagan T., Brewster M., Kesisoglou F., Beato S., Biewenga J., Crison J., Holm R., Li R., Mannaert E. (2017). A survey on IVIVC/IVIVR development in the pharmaceutical industry—Past experience and current perspectives. Eur. J. Pharm. Sci..

[34] Kambayashi A., Blume H., Dressman J.B. (2014). Predicting the oral pharmacokinetic profiles of multiple-unit (pellet) dosage forms using a modeling and simulation approach coupled with biorelevant dissolution testing: Case example diclofenac sodium. Eur. J. Pharm. Biopharm..

[35] Kambayashi A., Blume H., Dressman J. (2013). Understanding the in vivo performance of enteric coated tablets using an in vitro-in silico-in vivo approach: Case example diclofenac. Eur. J. Pharm. Biopharm..

[36] Schwalfenberg G.K. (2012). The alkaline diet: Is there evidence that an alkaline pH diet benefits health?. J. Environ. Public Health.

[37] Dissolution Method: Amoxicillin. https://www.accessdata.fda.gov/scripts/cder/dissolution/dsp_SearchResults.cfm.

