# Supplementary material for: Guiding a Diclofenac Sodium Dual-Release Sustained Formulation Development Through In Vitro–In Vivo Relationship Based on Physiologically Based Pharmacokinetics
Source: Pharmaceutics. 2026 May 18;18(5):613. doi: 10.3390/pharmaceutics18050613 (PMC13210601; doi:10.3390/pharmaceutics18050613)
Supplement: Supplementary file 1 [file pharmaceutics-18-00613-s001.zip › pharmaceutics-4258387-supplementary.pdf]

Guiding a diclofenac sodium dual-release sustained formulation development through in vitro-in vivo relationship based on physiologically based pharmacokinetics

Qizheng Wang, Pengcheng Guo, Tianci Hu, Longjie Li, Yue Pan, Xiaoqiang Xiang and Jianxin Wang

Supplement Materials

Table S1. Calibration formulation and liner rangers of Diclofenac Sodium in plasma.

Calibration formulation	R^2	Range (ng/mL)
$A_{S/I}$=0.0029C+0.0832	0.9999	10-5800

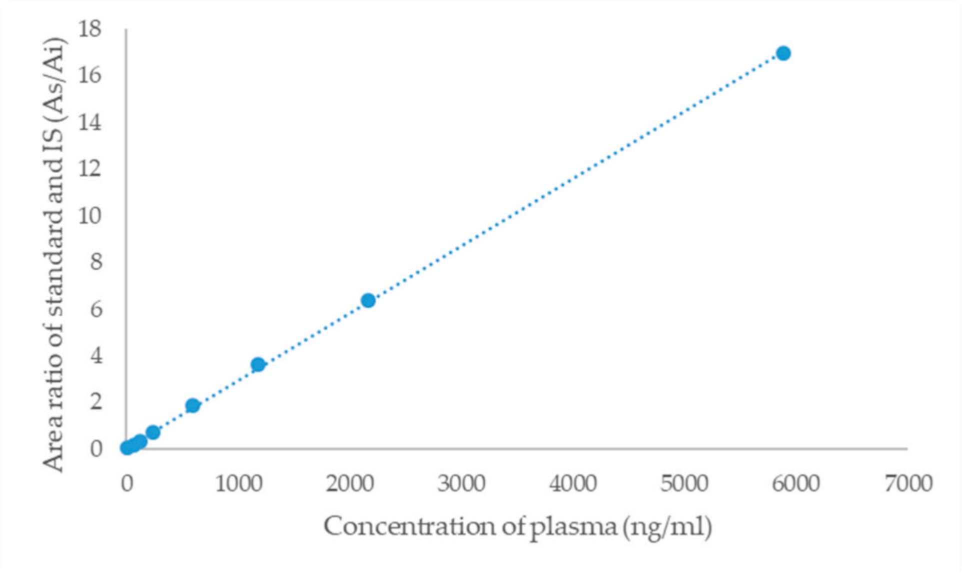

Figure S1. Calibration curve of Diclofenac Sodium in plasma.

Table S2. Recovery of Diclofenac Sodium and matrix effect in plasma. (n=6)

Concentration (ng/mL)	Diclofenac Sodium	Matrix effect
	Mean±RSD (%)	Mean±RSD (%)
50	106.69±5.09	103.79±6.38
200	100.96±3.75	99.31±4.11
2000	97.88±2.48	101.23±2.01

Table S3. Precision and accuracy of Diclofenac Sodium in plasma. (n=6)

	Concentration （ng/mL）	Mean±SD （%）	Precision （%）	Accuracy （%）
Intra-day	50	49.70±1.23	2.47	99.40
	200	201.32±9.55	4.74	100.66
	2000	1990.72±35.48	1.78	99.54

Inter-day	50	47.67±3.11	6.52	95.34
	200	194.63±7.75	3.98	97.32
	2000	1978.02±47.89	2.42	98.90

Table S4. Stability of Diclofenac Sodium in plasma. (n=6)

	Concentration （ng/mL）	Measured （ng/mL）	Relative Deviation （%）
Room temperature 24 h	50	49.42	-1.16
	200	200.18	0.09
	2000	2018.66	0.93
4 ℃ 24 h	50	52.07	4.14
	200	193.46	-3.27
	2000	2033.40	1.67
three freeze-thaw cycles	50	45.28	-9.44
	200	190.16	-4.92
	2000	1913.49	-4.33
